# Rethinking Coeliac Disease Diagnosis: Reflections on the 2025 ESsCD Guidelines

**DOI:** 10.1002/ueg2.70137

**Published:** 2025-10-22

**Authors:** Jason A. Tye‐Din

**Affiliations:** ^1^ Immunology Division The Walter and Eliza Hall Institute Parkville Victoria Australia; ^2^ Department of Medical Biology University of Melbourne Parkville Victoria Australia; ^3^ Department of Gastroenterology The Royal Melbourne Hospital Parkville Victoria Australia

The 2025 European Society for the Study of Coeliac Disease (ESsCD) guidelines on adult coeliac disease (CeD) diagnosis represent a major advance in clinical care [1]. The authors should be congratulated for preparing these comprehensive guidelines, which are timely and build upon the foundations of their 2019 predecessor [2]. To strengthen the consensus process, the Delphi method was employed in conjunction with the GRADE approach, ensuring methodological rigour and transparency. Significant effort was devoted to collating robust data and developing practical, evidence‐based algorithms that address common clinical scenarios, such as assessing villous atrophy in the absence of positive CeD serology. The multidisciplinary framework and integration of patient perspectives reflect an awareness of both practical challenges and future directions in CeD care. While topical, the field is at a crossroads: emerging immunological insights and practical challenges are reshaping our understanding of CeD and exposing the limitations of established diagnostic approaches [3].

Historically, CD has been viewed as a gut‐centric, malabsorptive illness, with small bowel histology as the diagnostic gold standard. The ESsCD 2025 guidelines maintain duodenal histology as the reference while, for the first time, conditionally permitting a no‐biopsy pathway for adults under 45 years with markedly elevated IgA anti‐TG2. The confirmation of a CeD diagnosis in adults can now, under specific circumstances, be based on positive serology alone (when the initial IgA anti‐TG2 level is at least 10 times the upper limit of normal and a second blood sample is positive). Decisions to omit endoscopy and biopsy and confirmation of the final diagnosis are to be made in secondary care and shared decision‐making with the patient is highlighted. This pragmatic move, finally echoing paediatric practice [4], acknowledges growing evidence for serological specificity and the need to reduce procedural burden. The diagnostic reliance on CeD serology underscores the importance of validated assays, and the guidelines advocate for certification, quality assurance, and standardisation through stakeholder collaboration and ongoing proficiency testing to ensure reliable global results.

With the recommendation of CeD serology as a standalone diagnostic tool in specific situations, the guidelines reflect an important shift towards immune blood‐based diagnostics that can confirm CeD without histology. Indeed, villous atrophy is neither universally present nor pathognomonic of CeD, and interobserver variability in histopathology remains problematic [3]. Quantitative histomorphometry, while more reliable, is seldom used outside research settings. This significant evolution away from a gut‐centric diagnostic focus may foreshadow the adoption of other immune diagnostic approaches. Indeed, novel biomarkers, such as interleukin‐2 (IL‐2), released after in vivo or in vitro gluten exposure, show promise for accurate CeD diagnosis without the need for a gluten challenge or biopsy, even in patients following a gluten‐free diet (GFD) [5, 6]. This approach sensitively detects gluten‐specific T cells, which are central to CeD pathogenesis [7]. Growing immune and genomic evidence reveals CeD is a systemic, T cell‐driven disorder, making T‐cell assays a logical diagnostic tool and suggesting it may soon be time to consider redefining CeD based on the presence of gut‐derived T‐cell responses to gluten rather than enteropathy [3]. This definition of CeD would support its modern conceptualisation as a systemic illness often with minimal enteropathy and potentially identify patients without overt enteropathy who benefit from a GFD [8]. Ongoing studies will determine the clinical utility and positioning of T‐cell diagnostic approaches.

Despite improvements in CeD detection, most CeD cases remain undiagnosed. The ESsCD guidelines support targeted screening of high‐risk groups, citing insufficient evidence for mass screening. However, population studies reveal that most individuals with CD do not have a positive family history or overt symptoms, and screen‐detected cases can benefit from treatment [9]. Ongoing trials in Europe and North America will clarify whether broader screening strategies can be justified by improved health outcomes and cost‐effectiveness.

The 2025 ESsCD guidelines provide practical recommendations for diagnosing CeD in adults on a GFD, specifying a minimum of 3 g/day gluten challenge for 6 weeks to balance accuracy and discomfort. This approach is pragmatic and makes the best use of limited controlled data and the absence of standardised gluten challenge protocols. The guidelines recommend initial HLA‐DQ2/DQ8 testing, emphasise shared decision‐making, and introduce useful strategies to minimise symptoms, such as low‐FODMAP gluten foods and distributing gluten intake throughout the day.

The ESsCD 2025 guidelines represent important progress in streamlining diagnosis and prioritising patient‐centred care in CeD. As the field evolves, future guidelines will need to incorporate emerging evidence, technologies, and patient experiences to further enhance outcomes. I look forward to the publication of Part 2 ESsCD guidelines, which will address CeD management and follow‐up.

## Conflicts of Interest

J.A.T.‐D. has privately or via his institute been a consultant or advisory board member for Anatara, Anokion, Barinthus Biotherapeutics, Chugai Pharmaceuticals, DBV Technologies, Dr Falk, EVOQ Therapeutics, Equillium, Forte Biosciences, IM Therapeutics, Janssen, Kallyope, Mozart Therapeutics, Sonoma Biotherapeutics, Takeda, TEVA and Topas, has received research funding from Barinthus Biotherapeutics, Chugai Pharmaceuticals, Codexis, DBV Technologies, Kallyope, Novoviah Pharmaceuticals, Topas and Tillotts Pharmaceuticals. He is an inventor on patents for the diagnosis and treatment of coeliac disease.

## Data Availability

The author has nothing to report.
